# Investigation of glutamine metabolism in CHO cells by dynamic metabolic flux analysis

**DOI:** 10.1186/1753-6561-7-S6-P44

**Published:** 2013-12-04

**Authors:** Judith Wahrheit, Averina Nicolae, Elmar Heinzle

**Affiliations:** 1 Biochemical Engineering Institute, Saarland University, D-66123 Saarbrücken, Germany

## Background

Glutamine metabolism represents one of the major targets in metabolic engineering and process optimization due to its importance as cellular energy, carbon and nitrogen source. Metabolic flux analysis represents a powerful method to investigate the physiology and metabolism of cells [1]. Classical metabolic flux analysis methods require steady state conditions. However, industrially relevant cultivation conditions, i.e. batch and fed-batch cultivations, are characterized by changing environmental conditions and metabolic shifts [2]. We used dynamic metabolic flux analysis to study the impact of glutamine availability or limitation on the physiology of CHO K1 cells capturing metabolic dynamics during batch- and fed-batch cultivations.

## Materials and methods

### Cell cultivation

CHO-K1 cells were cultivated in protein free TC-42 medium (TeutoCell, Bielefeld, Germany) in 50 ml filter-tube bioreactors (TPP, Trasadingen, Switzerland) at a start cell density of 2 × 10^5 ^cells/ml and a start volume of 20 ml. Cell counting and viability determination was carried out using an automated cell counter (Invitrogen, Darmstadt, Germany). Quantification of glucose, organic acids and amino acids via HPLC was carried out as described recently [3]. Ammonia was quantified using an ammonia assay kit (Sigma-Aldrich, Steinheim, Germany) in 96-well plates. Six different batch cultivations with 0 mM, 1 mM, 2 mM, 4 mM, 6 mM or 8 mM glutamine start concentration and two different fed-batch cultivations starting at 1 mM glutamine and feeding 1 mM every 24 h or starting at 2 mM and feeding 2 mM every 48 h were performed.

### Metabolic flux analysis

Splines were fitted to the cell density and the extracellular metabolite profile using the SLM curve fitting tool in Matlab 2012b (The Mathworks, Natick, MA, USA). Using a stoichiometric model of the CHO metabolism the intracellular fluxes were calculated by flux balancing.

## Results

Glutamine has an initial growth stimulating effect. With increasing glutamine concentration, the specific growth rate was initially higher but dropped earlier. However, increased accumulation of waste products at high glutamine levels, e.g. ammonia, inhibited growth later on and decreased culture longevity. The highest viable cell densities were reached in the 1 mM glutamine batch and 8 × 1 mM glutamine fed-batch cultivations.

Substantial dose-dependent flux rearrangements were observed for different glutamine availabilities. Initially, no significant impact on the glycolytic fluxes and lactate excretion was found. In later phases, glycolytic and lactate excretion rates were higher in the glutamine free cultivation. Waste product excretion of ammonia, alanine and glutamate increased with increasing glutamine concentration. The highest glutamate excretion was, however, found in the glutamine free cultivation. Uptake of pyruvate and serine as well as their importance as substrates increased with decreasing glutamine concentration and were highest under glutamine free conditions. This was accompanied by increasing excretion rates for glycine. At high glutamine concentrations, glutamate is converted to α-ketoglutarate feeding TCA cycle fluxes from α-ketoglutarate to oxaloacetate. However, due to an increased flux from oxaloacetate to phosphoenol pyruvate, fluxes from oxaloacetate to α-ketoglutarate were only significantly increased at 8 mM glutamine, but not at lower glutamine levels. The flux from oxaloacetate to phosphoenol pyruvate was reversed (phosphoenol pyruvate to oxaloacetate) at glutamine free conditions, resulting in anaplerotic feeding of carbon into the TCA cycle. The glutamate dehydrogenase flux was reversed (α-ketoglutarate to glutamate) at glutamine free conditions to produce glutamate for glutamine synthesis. Waste product excretion was reduced in the fed-batch cultivations compared to respective batch cultivations with 1, 2, or 8 mM glutamine. TCA cycle fluxes decreased over time in cultivations with high glutamine start concentrations and increased for cultivations with low initial glutamine levels and the glutamine free cultivation. With glutamine feeding, less variation of TCA cycle fluxes was observed.

## Conclusions

Dynamic metabolic flux analysis is a suitable method to describe the dynamics of growth and metabolism during batch and fed-batch cultivations with changing environmental conditions. For the batch cultivations, we observed dose-dependent effects of 1 to 8 mM glutamine start concentration. The fed-batch cultivations showed an intermediate response. The glutamine free cultivation had a very different physiology. Feeding of glutamine resulted in a reduced waste product excretion compared to respective batch cultivations and TCA cycle fluxes showed less variation during the cultivation process.
